# Membrane Remodeling as a Key Player of the Hepatotoxicity Induced by Co-Exposure to Benzo[a]pyrene and Ethanol of Obese Zebrafish Larvae

**DOI:** 10.3390/biom8020026

**Published:** 2018-05-14

**Authors:** Muhammad Imran, Odile Sergent, Arnaud Tête, Isabelle Gallais, Martine Chevanne, Dominique Lagadic-Gossmann, Normand Podechard

**Affiliations:** Inserm, EHESP, Irset (Institut de recherche en santé, environnement et travail)—UMR_S 1085, University of Rennes, F-35000 Rennes, France; muhammad.imran@univ-rennes1.fr (M.I.); odile.sergent@univ-rennes1.fr (O.S.); arnaud.tete@hotmail.fr (A.T.); isabelle.gallais@univ-rennes1.fr (I.G.); martine.chevanne@univ-rennes1.fr (M.C.); dominique.lagadic@univ-rennes1.fr (D.L.-G.)

**Keywords:** membrane remodeling, lipid raft, zebrafish larva, high-fat diet, liver steatosis, steatohepatitis, co-exposure, ethanol, benzo[a]pyrene, pravastatin

## Abstract

The rise in prevalence of non-alcoholic fatty liver disease (NAFLD) constitutes an important public health concern worldwide. Including obesity, numerous risk factors of NAFLD such as benzo[a]pyrene (B[a]P) and ethanol have been identified as modifying the physicochemical properties of the plasma membrane in vitro thus causing membrane remodeling—changes in membrane fluidity and lipid-raft characteristics. In this study, the possible involvement of membrane remodeling in the in vivo progression of steatosis to a steatohepatitis-like state upon co-exposure to B[a]P and ethanol was tested in obese zebrafish larvae. Larvae bearing steatosis as the result of a high-fat diet were exposed to ethanol and/or B[a]P for seven days at low concentrations coherent with human exposure in order to elicit hepatotoxicity. In this condition, the toxicant co-exposure raised global membrane order with higher lipid-raft clustering in the plasma membrane of liver cells, as evaluated by staining with the fluoroprobe di-4-ANEPPDHQ. Involvement of this membrane’s remodeling was finally explored by using the lipid-raft disruptor pravastatin that counteracted the effects of toxicant co-exposure both on membrane remodeling and toxicity. Overall, it can be concluded that B[a]P/ethanol co-exposure can induce in vivo hepatotoxicity via membrane remodeling which could be considered as a good target mechanism for developing combination therapy to deal with steatohepatitis.

## 1. Introduction

The significant rise in obesity prevalence in recent decades constitutes an important public health concern worldwide. It exposes a person to several pathophysiological ailments including steatosis defined by an excessive lipid accumulation in hepatocytes [1]. Steatosis dominates liver diseases in countries consuming the western diet, i.e., containing an important amount of fat and/or carbohydrates [2,3]. It is viewed as a benign hepatic lesion but can sensitize hepatocytes towards subsequent aggressions, thereby leading to steatohepatitis, which is characterized by liver cell death, inflammation and recurrent involvement of oxidative stress [4,5,6,7,8,9,10,11]. Furthermore, steatohepatitis can manifest in more severe hepatic diseases like fibrosis, cirrhosis and ultimately hepatocellular carcinoma (HCC) [12]. Hence, people with steatosis constitute a high-risk population for their evolution towards severe hepatic pathologies. In this context, more thorough research, notably regarding the factors driving the pathological progression of steatosis to steatohepatitis, is urgently needed.

Depending on the etiology, fatty liver diseases can be grouped in two categories: alcoholic liver diseases (ALD) and non-alcoholic fatty liver diseases (NAFLD) with a cut-off based on alcohol consumption of 20 g/day [13,14,15]. Considering NAFLD, beyond classical causes like lack of exercise, genetic predisposition, over-nutrition of fat/carbohydrates and associated obesity, a number of environmental toxicants has more recently been identified as implicated in similar liver diseases notably by affecting hepatic lipid metabolism, thus raising the concept of toxicant-associated fatty liver diseases (TAFLD) and toxicant-associated steatohepatitis (TASH) [16,17,18,19]. In addition, light or moderate alcohol consumption has also been raised as a possible factor of NAFLD even if it is still controversial [13,20]. Overall, obesity, alcohol and environmental contaminants—i.e., frequently involved factors in NAFLD—have been largely studied independently. Nevertheless, a few studies have shown prompt deterioration of liver state if two factors act simultaneously [13,21,22,23,24,25,26]. However, the effects of all three factors together on the liver state has rarely been explored to date [27]. In this context, the impact of the environmental contaminant, benzo[a]pyrene (B[a]P), in combination with an important lifestyle hepatotoxicant, ethanol, considering the obese (vulnerable) population frequently bearing hepatic steatosis, was described in a recent work by our team [27]. We showed that the association of these three different factors modeling these etiologies i.e., co-exposure to ethanol and B[a]P in animals fed with a high-fat diet (HFD) could drive liver disease progression [27]. B[a]P—an agonist of the aryl hydrocarbon receptor (AhR)—belongs to the polycyclic aromatic hydrocarbon family, and is a well-known genotoxic carcinogen for humans. It is a widespread environmental pollutant, which derives from diesel exhaust fumes, grilled food, cigarette smoke among other causes; it is biotransformed by liver, and it is suggested that it induces liver steatosis and HCC, not only in experimental models but also in humans [22,28,29,30].

During the last few years, various mechanisms responsible for chemical-induced hepatotoxicity have been suggested. Among them, in recent times, membrane remodeling—defined as changes in membrane fluidity and/or in lipid raft characteristics—have been identified as a common toxic mechanism for several chemicals, including B[a]P and ethanol, both in vitro and in vivo [31,32,33,34,35]. In fact, it has been shown that B[a]P can activate the cytosolic receptor AhR with a consequent translocation of the ligand–receptor complex to the nucleus; after heterodimerization with its partner, the aryl receptor nuclear translocator. AhR could then act as a transcription factor. This AhR activation along with reactive oxygen species (ROS) production—linked to the metabolism of B[a]P—could affect lipid metabolism, thereby decreasing cholesterol synthesis and inducing membrane remodeling; such remodeling was responsible for hepatocyte death in vitro [30,36,37]. Ethanol-induced membrane remodeling is also largely reported to be involved in hepatocyte toxicity and in liver injury both in vitro and in vivo, notably through toll-like receptors (TLR) activation, which are proteins described as located in plasma membrane lipid rafts of liver cells [33,34,35,38,39]. Furthermore, our team has also described that, in vitro, membrane remodeling can play a key role in cell death induced by B[a]P/ethanol co-exposure of non steatotic hepatocytes, even at low doses [40]. In addition, a role for membrane remodeling is asserted in non-alcoholic steatohepatitis linked to obesity and HFD, as TLRs have been identified as key players of this disease [4,41]. Finally, involvement of membrane remodeling in chemical-induced in vivo hepatotoxicity has been shown in a model of a zebrafish larva [33]. However, even if we have found that co-exposure to low doses of B[a]P and ethanol could drive the progression of HFD-induced steatosis to a steatohepatitis-like state in this model [27], the involvement of membrane remodeling in this multifactorial NAFLD progression has not been explored yet.

To this end, we have focused our study on the model of zebrafish larva for several reasons. Zebrafish and humans largely share genomic homology. These animals exhibit rapid but similar liver development to rodents and humans, with which they share common physio-pathological processes [42,43,44,45]. From 5 days post-fertilization (dpf), the liver is indeed functional and expresses enzymes responsible for xenobiotic metabolism that resemble those expressed in humans like cytochrome P450 2E1 (CYP2E1) and alcohol dehydrogenase for alcohol or CYP1A for B[a]P [43,46,47,48,49]. Beside the numerous advantages of zebrafish larva, notably for assessing hepatotoxicant effects [50,51,52,53], this model has already been demonstrated as very useful for studying fatty liver diseases [54,55,56,57,58,59]. In addition, the suitability of this model for studying the involvement of membrane remodeling in chemical-induced liver toxicity has already been reported [33]. Finally, we have recently described that co-exposure of HFD-fed larva to B[a]P and alcohol leads to a steatohepatitis-like state of the liver [27].

In the present study, the objective was, thus, to test the possible involvement of membrane remodeling in the disease progression of steatosis in zebrafish HFD-fed larvae upon co-exposure to two second hits, that is B[a]P and ethanol, at low doses. Therefore, at first, we evaluated steatosis with a Nile red staining; then its evolution towards steatohepatitis-like state after seven days of co-exposure was studied by examination of a histological liver injury and an assessment of the expression of several genes involved in the characteristic features of steatohepatitis—inflammation, cell death, markers of hepatotoxicity and general cellular stress response. In a second part, we evaluated membrane remodeling by staining with the fluoroprobe di-4-ANEPPDHQ, which is sensitive to membrane order. Finally, the involvement of membrane remodeling in this pathological progression of steatosis was explored by using pravastatin—a drug described for its capacity to disturb membrane properties, especially at the lipid-raft level through the inhibition of endogenous cholesterol synthesis.

## 2. Results

### 2.1. Progression of High-Fat Diet Induced Steatosis to a Steatohepatitis-Like State in Zebrafish Larvae upon Co-Exposure to B[a]P and Ethanol

**Figure 1:**
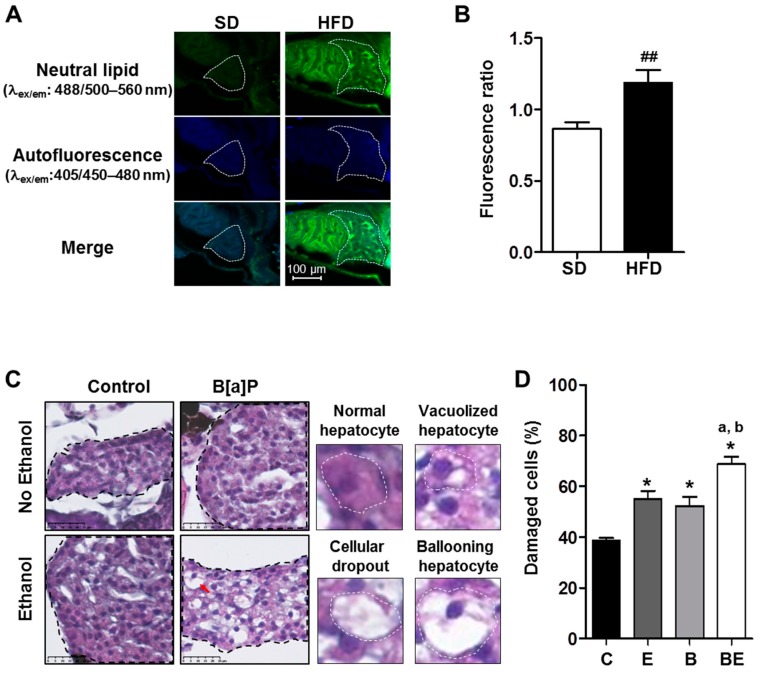
Progression of high-fat diet (HFD) induced steatosis in zebrafish larvae to a steatohepatitis-like state upon co-exposure to ethanol and benzo[a]pyrene. Zebrafish larvae were fed with a HFD from 4 days post-fertilization (dpf) until 5 dpf and compared to larvae fed with a standard diet (SD) in order to observe the development of steatosis at 5 dpf (**A**,**B**). Lipid accumulation was analyzed after Nile red staining in HFD larvae as well as in SD larvae using confocal microscopy (excitation/emission (ex/em) wavelength: 488/500–560 nm, magnification ×400). (**A**) Representative images of larva staining are presented in which the liver has been outlined in white. (**B**) In order to estimate the relative amount of neutral lipids in the liver, the ratio of fluorescence intensity was calculated from images of more than 15 larvae per diet as follows: Fluorescence ratio = (intensity of neutral lipid staining with Nile red (ex/em wavelength: 488/500–560 nm)/(intensity of unspecific staining (autofluorescence; ex/em wavelength: 405/450–480 nm))). Values are the mean ± standard error of the mean (SEM) of at least 12 larvae per diet. Zebrafish larvae fed with HFD from 4 dpf and exposed to ethanol and/or B[a]P for seven days from 5 to 12-dpf to achieve four conditions—untreated (C) or treated with 25 nM B[a]P (B), 43 mM ethanol (E) or a combination of both toxicants (BE,C,D). (**C**) Liver damage was evaluated on zebrafish liver sections after HES staining (magnification ×400). Black dotted line outlines liver. Histological liver sections were magnified to show, surrounded by the white dotted line, a normal hepatocyte, a vacuolized hepatocyte, a cellular dropout and a ballooning hepatocyte (red arrow). Images are representative of at least five larvae. (**D**) From images obtained in (**C**), the histological count of damaged cells was realized. Values are the mean ± SEM of at least five larvae. ^##^ Significantly different from SD larvae; * Significantly different from HFD control larvae; ^a^ Significantly different from larvae treated by ethanol only; ^b^ Significantly different from larvae treated by B[a]P only.

We previously developed an in vivo zebrafish larva model with or without steatosis for studying the effects of various toxicants [27,33]. We found that liver steatosis could be induced in zebrafish larvae at 5 dpf, with only one day of HFD that increased oil red o staining, liver size with respect to whole body, triglyceride content, and mRNA level of apolipoprotein A-II (*apoa2* and *cyp2y3* gene—homologous of the human *CYP2E1* gene—in comparison to a standard diet (SD) [27]. In the present study, steatosis was further confirmed by using Nile red staining. The fluorescence ratio of the stained liver of HFD-fed larvae was, indeed, significantly higher compared to the SD-fed larvae liver (Figure 1A,B), thus indicative of an accumulation of neutral lipids in the liver of HFD-fed larvae.

Following the onset of steatosis, larvae were then exposed to ethanol or B[a]P alone or in co-exposure at sub-lethal concentrations for seven days in order to elicit pathological progression of this disease. For each toxicant, a dose of exposure was chosen in respect to the human level of exposure. Thus, the dose used for ethanol was 43 mM that reached 10 mM (0.46 g/L) inside larvae (data not shown). This concentration is less than drinking guidelines for general populations issued by the International Alliance for Responsible Drinking in 2017 [60]. Considering B[a]P, a concentration range of 0.5–40 nM was obtained in serum from military personnel [61]. Thus, the dose of B[a]P, selected for the present study, was 25 nM. Liver cell damage—a prime characteristic of a progression towards steatohepatitis [6,11,62]—was analyzed by producing histological liver sections of zebrafish larva (Figure 1C), and damaged hepatocytes—ballooning cells, vacuolated cells and hepatocyte dropouts—were counted in each experimental condition. As shown in the histogram (Figure 1D), ethanol and B[a]P alone enhanced liver toxicity as visualized by an increased number of damaged cells in comparison to the control, with a further significant effect of co-exposure compared to all other conditions.

**Figure 2:**
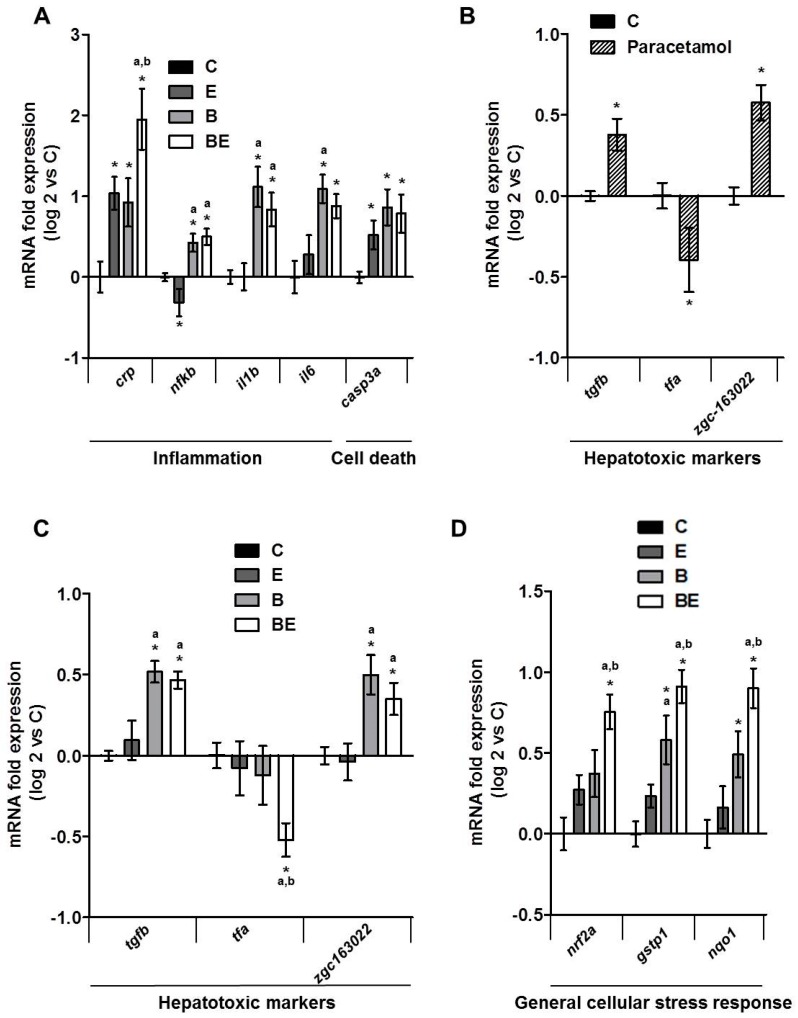
Impact of B[a]P/ethanol co-exposure on the mRNA expression of several genes involved in different biological processes characteristic of steatohepatitis. mRNA expression was evaluated by quantitative reverse transcription polymerase chain reaction (RT-qPCR) (**A**–**D**). Zebrafish larvae were fed with HFD from 4 dpf and exposed to ethanol and/or B[a]P for seven days from 5 to 12 dpf to achieve four conditions—untreated (C) or treated with 25 nM B[a]P (B), 43 mM ethanol (E) or a combination of both toxicants (BE). For the experiments with paracetamol, 1 mM paracetamol was added to the incubation medium containing zebrafish larvae from 5 to 12 dpf. mRNA expression of genes characteristic of inflammation and cell death (**A**), hepatotoxicity (**B**,**C**) and general cellular stress response (**D**) are shown. Data are expressed relative to mRNA levels found in HFD control larvae, set at 0 (log 2 change). Values are the mean ± SEM. * Significantly different from HFD control larvae; ^a^ Significantly different from larvae treated by ethanol only; ^b^ Significantly different from larvae treated by B[a]P only.

The second main characteristic of steatohepatitis—i.e., inflammation [6,7,8,9,10,11,62]—was tested by analyzing the mRNA expressions of various inflammatory gene markers, such as *crp*, *nfkb*, *il1b* and *il6*, in whole larvae (Figure 2A). mRNA expressions of all four inflammatory markers were significantly higher in larvae co-exposed with B[a]P and ethanol in comparison to control larvae with a more marked effect regarding *crp*. Ethanol or B[a]P alone were also able to induce these expressions but a further significant induction was observed on *crp* expression when larvae were exposed to both toxicants.

The cell death marker, *casp3a*, was also found to increase significantly in toxicant co-exposed larvae, which is coherent with a classical increase of apoptosis in steatohepatitis [63,64] (Figure 2A). However, a similar increase as for co-exposure was also observed with each toxicant alone. Several studies have described markers characteristic of hepatotoxicity in zebrafish such as *tgfb*, *tfa*, *zgc163022* in addition to *nfkb*, *casp3a* and some other [50,52]. For validation of these hepatotoxic markers in our conditions, we treated zebrafish larvae with paracetamol (1 mM), a well-known hepatotoxic agent, for the same long-term exposure as for B[a]P and ethanol and tested the expression of markers representative of hepatotoxicity (Figure 2B). Our data clearly indicated that paracetamol significantly altered the expression of these hepatotoxic markers with a rise of *tgfb* and *zgc163022* and an inhibition of *tfa* expression as already described for short-term exposure [50,56]. Therefore, we decided to quantify the mRNA expression of these hepatotoxic markers in larvae co-exposed with ethanol and B[a]P. As illustrated in Figure 2C, a significant change in expression of *tgfb*, *tfa* and *zgc163022* was observed, in a similar way as with paracetamol, especially in co-exposed larvae. This thus confirmed the hepatotoxicity of the B[a]P/ethanol co-exposure. Finally, the expression of genes induced in response to general cellular stress, such as *nrf2a*, *nqo1* and *gstp1,* was also tested since cellular stress is commonly associated with NAFLD and xenobiotic metabolism/toxicity [65,66]. We found that the expression of these genes was significantly augmented with either toxicant (Figure 2D). In coherence with *crp*, co-exposure further enhanced, in a significant manner, the expression of all three genes induced in response to general cellular stress in comparison to toxicants alone (Figure 2D). Together, these results confirm that co-exposure to both toxicants drives the progression of steatosis toward a steatohepatitis-like state even if further investigation will be required to fully confirm the inflammatory state, notably by looking for immune cell infiltration.

### 2.2. Involvement of Membrane Remodeling in the Hepatotoxicity Induced by B[a]P and Ethanol Co-Exposure in Zebrafish Larvae

**Figure 3:**
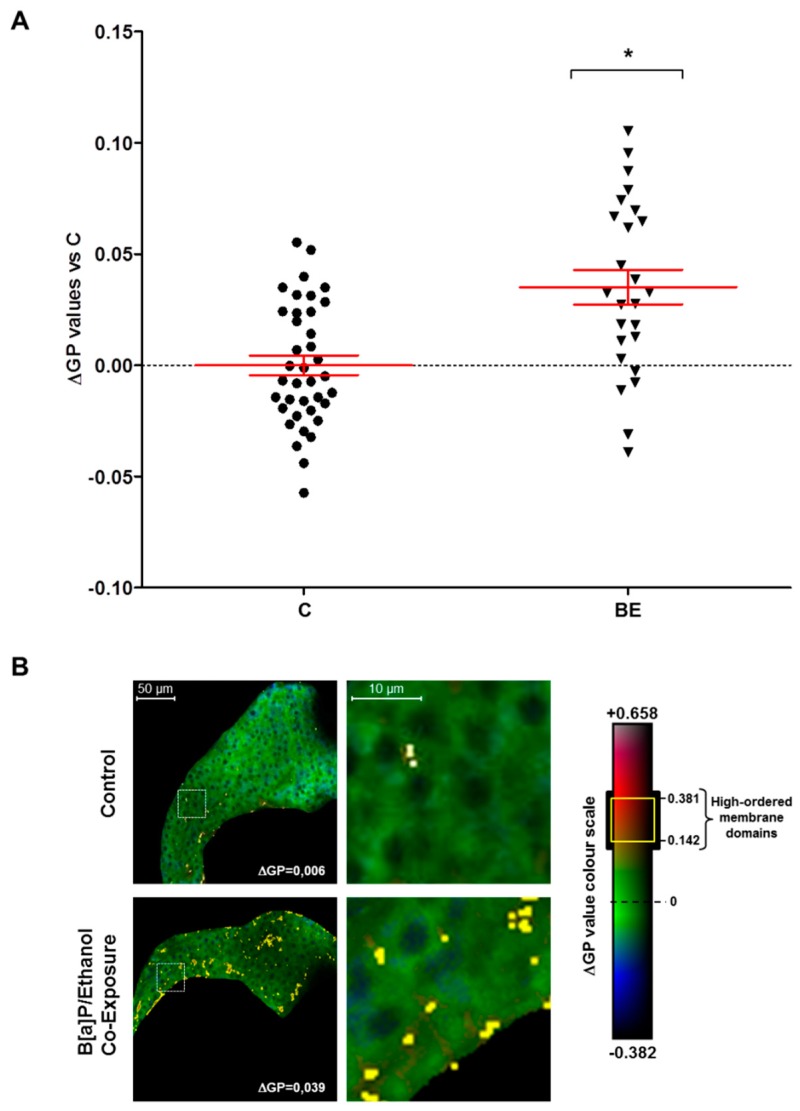
Co-exposure to alcohol and benzo[a]pyrene-induced membrane remodeling in the liver of HFD zebrafish larvae. Membrane order and lipid raft spatial distribution characteristics of membrane remodeling was assessed in liver cells of steatotic zebrafish larvae after co-exposure to ethanol and B[a]P for seven days from 5 to 12 dpf. Zebrafish larvae under two conditions—untreated (C) or treated with combination of 43 mM ethanol and 25 nM B[a]P (BE)—were stained with di-4-ANEPPDHQ—a membrane order-sensitive fluorescent probe—and analyzed by confocal fluorescence microscopy. Membrane order in membranes of zebrafish liver was measured by computing the generalized polarization (GP) factor. (**A**) Changes in GP values were expressed as the difference between individual larva GP value and the mean of GP found in control larvae (ΔGP). (**B**) On the left, some representative liver images of each treatment have been selected according to the respective mean of delta GP (magnification ×400). Pixels with higher GP values (which could be considered as lipid rafts) have been highlighted in yellow to pinpoint lipid raft spatial distribution. The liver area outlined in the white square on the left images are magnified on the right side to show lipid raft spatial distribution in the plasma membrane. Values are the mean ± standard error of the mean (SEM) of at least 25 larvae. * Significantly different from HFD control larvae.

Previously, our team described the reliability of the zebrafish model to study the effects of hepatotoxicants on plasma membranes [33]. Further, membrane remodeling was identified as a key mechanism of co-exposure to B[a]P and ethanol to induce hepatotoxicity in vitro [40] and for ethanol in vivo [33]. However, the involvement of membrane remodeling in vivo has never been investigated in the context of steatosis progression, notably upon co-exposure with B[a]P and ethanol. In the present study, the impact of such a co-exposure on membrane remodeling was thus determined by analysis of membrane order with the fluorescent hydrophobic probe—di-4-ANEPPDHQ. This allowed us to calculate a generalized polarization (GP) value representative of membrane order that depends on the chemical and physical properties of membranes—lipid composition and packing, fluidity and lipid bilayer thickness, and local hydration. In addition, lipid rafts—specialized membrane microdomains that can also be defined by their high membrane order—could be visualized through membrane areas with high GP values [33,67,68,69]. After staining the whole zebrafish larvae with di-4-ANEPPDHQ, liver images—characteristic of membrane order—were acquired by computing the GP value obtained from fluorescence images of lipid bilayers with low-membrane lipid order—the liquid disordered (L_d_) phase—and with high-membrane lipid order—the liquid ordered (L_o_) phase. It was observed that exposure of zebrafish larvae to ethanol or B[a]P alone has no significant effect on global membrane order in liver cells (data not shown). However, when tested in combination, they enhanced global membrane order in a significant manner in the liver of HFD-fed zebrafish larvae (Figure 3A). Furthermore, numerous membrane domains with high GP value-defining lipid rafts, possibly reflecting their clustering, were observed at the level of the plasma membrane of liver cells in larvae treated with toxicant co-exposure in comparison to untreated larvae (Figure 3B). This, therefore, indicated that B[a]P/ethanol co-exposure induced membrane remodeling in HFD-fed larvae.

### 2.3. Role for Membrane Remodeling in the Protective Effect of Pravastatin against Co-Exposure-Induced Hepatotoxicity in Zebrafish Larvae

**Figure 4:**
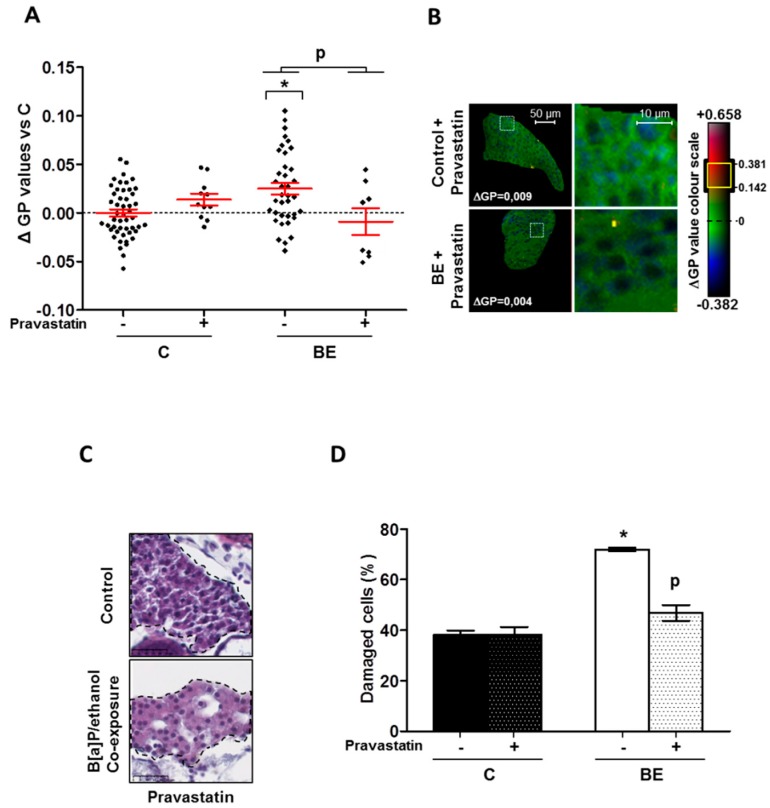
Protective effect of pravastatin towards membrane remodeling and hepatotoxicity-induced by B[a]P/ethanol in HFD zebrafish larvae. Membrane remodeling was assessed in the liver of HFD steatotic zebrafish larvae after exposure to ethanol and B[a]P for seven days with and without pravastatin (0.5 µM) from 5 to 12 dpf. Zebrafish larvae under four conditions, control (untreated (C) ± Pravastatin), or treated with combination of both toxicants (BE ± Pravastatin; 25 nM B[a]P and 43 mM ethanol) were stained with di-4-ANEPPDHQ—a membrane order-sensitive fluorescent probe—and analyzed on confocal fluorescence microscopy. Membrane order in membranes of zebrafish liver was measured by computing GP factor. (**A**) Changes in GP values were expressed as the difference between individual larva GP value and the mean of GP found in control larvae (ΔGP). Values are the mean ± SEM of at least eight larvae. (**B**) On the left, some representative liver images of each treatment have been selected according to the respective mean of delta GP (magnification ×400). Pixels with higher GP values (could be considered as lipid rafts) have been highlighted in yellow through membrane area of liver cells to pinpoint lipid raft spatial distribution. Liver area outlined in white square on left images are magnified on right side to show lipid raft spatial distribution in plasma membrane. (**C**) Liver damages were evaluated on zebrafish liver section after HES staining (magnification ×400). Black dotted line outlines liver. Images are representative of at least 3 larvae. (**D**) From images obtained in (**C**), histological count of damaged cells was realized. Values are the mean ± SEM of at least three larvae. * Significantly different from HFD control larvae; ^P^ Significant difference between larvae treated by pravastatin compared to untreated counterparts.

**Figure 5:**
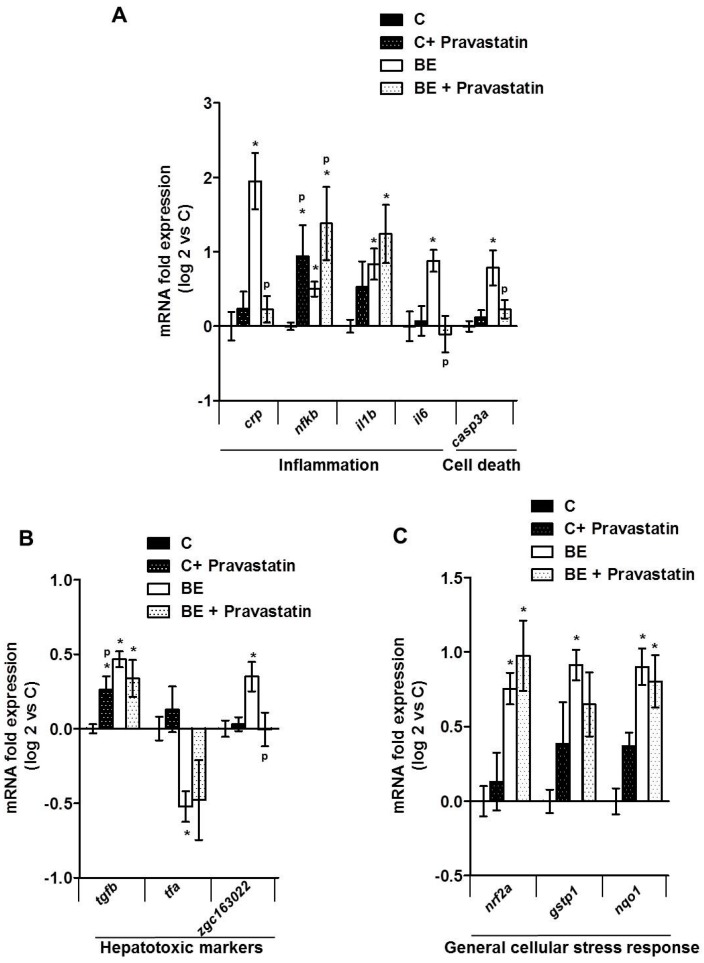
Impact of pravastatin on mRNA expression of several genes involved in different biological processes characteristic of steatohepatitis after exposing HFD zebrafish larvae to a combination of B[a]P and ethanol. mRNA expressions of several genes were evaluated by RT-qPCR (**A**–**C**). Zebrafish larvae were fed with HFD from 4 dpf and from 5 dpf, they were either left untreated (C) or treated with co-exposure of 43 mM ethanol and 25 nM B[a]P (BE) until 12 dpf. Both conditions were also treated with 0.5 µM pravastatin as quoted as (C ± pravastatin) and (BE ± pravastatin), respectively. mRNA expressions of genes characteristic of inflammation and cell death (**A**), hepatotoxicity (**B**) and general cellular stress response (**C**) are shown. Data are expressed relative to mRNA level found in HFD control larvae, set at 0 (log 2 change). Values are the mean ± SEM. * Significantly different from HFD control larvae; ^P^ Significant difference between larvae treated by pravastatin compared to untreated counterparts.

Finally, with the aim of testing the involvement of membrane remodeling in hepatotoxicity produced by B[a]P/ethanol co-exposure, a lipid raft disrupter, pravastatin [70], was used as it was demonstrated to be effective in zebrafish larvae [33] (Figure S1). Pravastatin—a cholesterol synthesis inhibitor—prevented the effects of co-exposure to B[a]P and ethanol on the cell membrane in zebrafish larvae by significantly reducing the membrane order (Figure 4A). Moreover, it also decreased the impact of co-exposure on the lipid raft spatial distribution in the plasma membrane of liver cells, thus pointing to a prevention of lipid-raft clustering (Figure 4B). Regarding the consequences in terms of hepatotoxicity, the histological analysis of liver from steatotic zebrafish larvae co-exposed with pravastatin and toxicants showed less liver cell damage (Figure 4C) compared to larvae unexposed to pravastatin (Figure 1C). Indeed, this molecule significantly reduced the number of damaged cells (Figure 4D). The last set of experiments was performed to test the impact of pravastatin on the mRNA expression of the genes altered by B[a]P/ethanol co-exposure. Our data showed that pravastatin prevented alterations in the expression of several genes involved in inflammation (*crp*, *il6*), cell death (*casp3a*) and also one hepatotoxic marker (*zgc163022)*. However, no effect on *tfa* and on genes related to cellular stress response (*nrf2a*, *nqo1* and *gstp1*) was detected (Figure 5A–C). Note that pravastatin alone enhanced the expression of *nfkb*, *il1b* and, to a lesser extent, *tgfb* (Figure 5A,B). Overall, our results indicated that pravastatin could protect liver from injury induced by toxicant co-exposure, thus indicating the involvement of membrane remodeling and especially lipid-raft clustering in the pathological progression of steatosis upon co-exposure to B[a]P and ethanol.

## 3. Discussion

Several mechanisms are known to be involved in the toxicity of B[a]P and ethanol towards the liver—oxidative stress, cell death, inflammation and mitochondrial dysfunction [33,36,40,71,72,73,74]. In addition, another process that has been highlighted in this context is membrane remodeling. In fact, B[a]P was suggested to repress HMGCR (3-hydroxy-3-methylglutaryl-CoA reductase) in vitro via AhR activation and ROS, thus hindering cholesterol synthesis and modulating the lipid content of lipid rafts, finally leading to hepatocyte cell death [36]. Ethanol is also described to alter cell membrane properties by modifying fluidity and lipid-raft clustering in plasma membrane in vitro and in vivo with consequences on cell death and liver injury [33,38]. More recently, co-exposure to B[a]P and ethanol was also shown in vitro in primary hepatocytes to induce membrane remodeling, with consequences in terms of hepatotoxicity [40]. Besides, these same compounds used in combination were demonstrated to induce the progression of steatosis to a steatohepatitis-like state, notably in a model of zebrafish larva. At the same time, HFD—the principal cause of steatosis—was also identified as modifying the physicochemical properties of the membrane by altering its lipid composition or lipid-raft protein activity; it was proposed that this process was involved in NAFLD progression in association or not with hepatotoxicants [41,75,76]. Therefore, in the present study, membrane remodeling was explored in vivo, using the steatotic zebrafish larva model co-exposed with B[a]P and ethanol in order to assess its implications for steatohepatitis development. We found that B[a]P and ethanol, when applied together, significantly altered zebrafish liver cell membrane properties by increasing the overall membrane order in comparison to the control. In parallel, a larger staining of high-ordered membrane domains—showing higher lipid-raft spatial distribution—was also seen in the plasma membranes of larvae liver cells when co-exposed with B[a]P and ethanol, thus emphasizing more lipid-raft clustering. This increase in membrane order and modification of lipid-raft spatial distribution—two indicators of membrane remodeling—were coherent with histological sections, showing more damaged hepatocytes in toxicant co-exposed larvae. However, exact characterization of membrane remodeling—global and local membrane fluidity and lipid-raft microdomain structures—still needs further investigation with special emphasis on B[a]P effects on cholesterol content and discrepancies over its effects in comparison to those observed in vitro [36,40]. Higher membrane remodeling and, notably, the higher level of plasma membrane lipid-raft clustering, suggest alteration of the lipid raft-associated signaling pathway. Several previous studies have proven that alterations in cell membrane properties can modulate several membrane receptors linked with lipid rafts such as toll-like receptors (TLR 2, 4 and 9), which induce, notably through the activation of NF-κB release, a variety of pro-inflammatory cytokines such as interleukins (IL-1β and IL-6) and TNFα [39,41,62,77,78]. Here, mRNA expression of several inflammatory and hepatotoxic markers was found to be increased including *il1b*, *il6* and *nfkb*. The simultaneous membrane remodeling, hepatocellular damage, and increase in inflammatory markers associated with lipid rafts, therefore, suggested a link between hepatotoxicity of co-exposure to B[a]P and ethanol and membrane remodeling.

The participation of membrane remodeling in co-exposure-induced hepatotoxicity was thus tested in zebrafish larvae by assessing the impact of pravastatin; this molecule is indeed a known lipid-raft disrupter. The addition of pravastatin along with B[a]P and ethanol counteracted the effects of toxicant co-exposure on membrane order (Figure 4A) and prevented changes in lipid raft spatial distribution (Figure 4B). Histological analysis then showed less hepatocyte damage, likely due to the protective action of pravastatin on the hepatocyte cell membrane. To confirm the role of membrane remodeling, we tested pravastatin in vitro on the WIF-B9 hepatic cell line—an in vitro model of well differentiated hepatocytes [33,79,80,81]. This cell line exhibited a similar type of results as in the zebrafish larvae model; indeed, pravastatin decreased the number of apoptotic cells induced by toxicant co-exposure in a steatotic state (Figure S2). These protective effects towards cell death were further supported by the fact that pravastatin in zebrafish larvae prevented the effects of co-exposure on the mRNA expression of *casp3a* and the hepatotoxic marker *zgc163022*. Besides the protection afforded towards cell death, this molecule also inhibited the increase in mRNA expression of inflammatory markers, namely *crp* and *il6*, in line with the previously described role for lipid rafts in steatohepatitis-related inflammation [41,77]. Altogether, these results, therefore, indicate that pravastatin would decrease the observed hepatotoxicity by counteracting membrane remodeling, thereby further endorsing the contribution of membrane remodeling as a key player in the pathological progression of steatosis induced by a mixture of toxicants such as B[a]P and ethanol. One might have argued that the protective effect would have been through induction of *cyp1a* expression or through a decrease of ethanol metabolism via inhibition of *cyp2y3* expression, the zebrafish homolog of *CYP2E1*, or through NRF2 pathway activation. Indeed, it has been previously reported that CYP1A1 can afford some protective action against NAFLD in dioxin- [82] or B[a]P-exposed mice [22]; besides, it is known that CYP2E1 is involved in ethanol toxicity [83], even in the zebrafish model [54]. Similarly, NRF2 pathway activation may also provide protection through xenobiotic metabolism or via an action against oxidative stress [84,85,86]. However, in our model, pravastatin had no significant effect on *cyp1a* or *cyp2y3* expression (supplementary Figure S3A,B) nor on *nrf2a* and its regulated genes (Figure 5C), thus further reinforcing a key role for membrane remodeling.

Although, pravastatin appeared to prevent hepatotoxicity, it seems that this protection would be only partial in our model; indeed the alterations observed in the mRNA expression of several genes were not all blocked. Henriksbo and Schertzer [87] have previously described the impact of pravastatin per se on inflammatory markers such as CRP, IL-1β and IL-6. Whereas they reported a decrease in CRP and IL-6 mRNA expression, which is clearly in favor of a protection afforded towards inflammation, they also found that pravastatin increased the expression of IL-1β. Quite a similar result was obtained in our study with an increased mRNA expression of *il1b* upon pravastatin; likewise, an increase in *nfkb* expression (another inflammatory marker) was detected. This increase in some inflammatory mediators/regulators might suggest exacerbation of inflammation in the liver and/or whole larvae by pravastatin. A relatively similar type of finding regarding the liver was previously observed with statins by others [88,89]. Such a proper effect of pravastatin might explain why no protective effect of this compound towards toxicant co-exposure impact on these genes could be observed (Figure 5A). Regarding the other genes studied, that is *tfa* and *tgfb*, both previously shown as hepatotoxic markers [50,56], no effect of pravastatin towards co-exposure effects was detected. As already mentioned, B[a]P and ethanol could produce hepatotoxicity via several mechanisms. Based upon our results, pravastatin via cholesterol synthesis inhibition appeared to prevent toxicant effects on membrane remodeling, which thus pointed to membrane remodeling as being involved in steatosis progression. However, even though such a mechanism would be involved, we cannot yet exclude other mechanisms for the action of statins such as an effect on mitochondrial fatty acid oxidation [90], SREBP-2 (sterol regulatory element binding transcription factor 2) induced autophagy [91], and others [92,93,94], to be effective in NAFLD [95,96,97,98,99].

Overall, this study shows for the first time that toxicant co-exposure can favor the progression of liver steatosis towards a steatohepatitis-like state by inducing membrane remodeling, which is involved in both cell death and inflammation. This mechanism can be switched off by a lipid-raft disrupter. Therefore, this mechanism could be considered as a good target in addition to other mechanisms—oxidative stress, inflammation, apoptosis and fibrosis [63]—for developing combination therapy to deal with steatohepatitis.

## 4. Materials and Methods

### 4.1. Zebrafish Larvae Handling and Exposure

Animals were handled, treated and killed in agreement with the European Union regulations concerning the use and protection of experimental animals (Directive 2010/63/EU). All protocols were approved by local ethic committee CREEA (Comité Rennais d’Éthique en matière d’Expérimentation Animale, Rennes, France; approval number R-2012-NP-01). Fertilized zebrafish embryos—collected following natural spawning—were obtained from the Structure Fédérative de Recherche Biosit (INRA LPGP, Rennes, France). Embryos and larvae—sex unknown—were raised at 28 °C according to standard procedures and as previously described [33]. From 4 dpf until the last day of treatment renewal—at 9 dpf—larvae were fed daily with a SD, 10% of fat (Tetramin, Tetra, Blacksburg, VA, USA), or with a HFD made of chicken egg yolk, ~53% of fat (Sigma-Aldrich, St. Louis, MO, USA), for 1 h before medium renewal. Both diets were also previously used in zebrafish [27,100,101]. At 5 dpf, larvae were exposed with 43 mM ethanol directly added to the incubation medium and/or by 25 nM B[a]P in dimethyl sulfoxide (DMSO)—DMSO final proportion: 0.001% *v/v*—or by this vehicle only until 12-dpf. For experiments with pravastatin, 0.5 μM pravastatin (Sigma-Aldrich), was added along with toxicants simultaneously; for experiments with paracetamol (1 mM; Acetaminophen; Sigma-Aldrich), this was added to the incubation medium.

### 4.2. Neutral Lipid Staining with Nile Red

At 5 dpf, after 24 h of feeding, zebrafish larvae were washed in phosphate buffered saline (PBS) and then fixed in 4% paraformaldehyde in PBS at 4 °C. A staining protocol of neutral lipids in liver with Nile red was adapted from previous works [59,102]. After washing in PBS, whole larvae were stained for 1 h with Nile red at 5 µg/mL (N3013, Sigma-Aldrich; stock solution was prepared at 100 µg/mL in acetone). Then, larvae were washed twice in PBS and mounted on slides with PBS. Images of zebrafish larvae were acquired with a confocal fluorescence microscope LEICA DMI 6000 CS (Leica Microsystems, Wetzlar, Germany). To evaluate neutral lipid content, a first image—characteristic of neutral lipid fluorescence—was taken under excitation at 488 nm using an argon ion laser with a photomultiplier tube (PMT) range of 500–560 nm (image A) whereas a second image—insensitive to neutral lipids—was taken under excitation at 405 nm with a diode laser with a PMT range of 450–480 nm (image B) (magnification ×400). Using Fiji imaging processing software(ImageJ, [103]), fluorescence intensity per liver area was calculated for both images; finally, the fluorescence ratio of image A to image B was determined.

### 4.3. Histological Analysis of Liver Toxicity in Zebrafish Larvae

Histological analysis was performed as previously described [27]. Briefly, after treatment, larvae at 12 dpf were washed in PBS and then fixed in 4% paraformaldehyde in PBS at 4 °C before being embedded in paraffin. Then, 5 μm sections were stained with hematoxylin, eosin and safran red (HES) and imaged on a Nanozoomer NDP (Hamamatsu Photonics K.K., Hamamatsu, Japan) (magnification ×400). A histological count of dead/damaged cells was performed from images (two or three sections) of at least three larvae per condition. Damaged/dead cells were counted as cellular dropouts [104], ballooning cells [105], and vacuolated hepatocytes [51].

### 4.4. Analysis of Gene mRNA Expression

Analysis of gene mRNA expression was performed as previously defined [27]. For mRNA extraction, 10–20 larvae were pooled and homogenized in 100 μL TRIzol reagent and total RNA was extracted according to the manufacturer’s protocol with TRIzol reagent. RNA samples (1 μg) were then reverse-transcribed using the High-Capacity cDNA Reverse Transcription Kit (Life Technologies, Carlsbad, CA, USA). Quantitative reverse transcription polymerase chain reaction (RT-qPCR) (5 ng of cDNA per well) was performed using SYBR Green on the CFX384 Touch Real-Time PCR Detection System (Bio-Rad, Hercules, CA, USA). mRNA expression was normalized by means of *actb2*, *18s* and *gapdh* mRNA levels. The ΔΔCt method was used to indicate the relative expression of each selected gene. Sequences of the tested zebrafish primers are provided in Table 1.

### 4.5. Membrane Order Determination by Fluorescence Staining

Plasma membrane order in zebrafish liver was assessed, as previously defined [33], by confocal fluorescence microscopy using the membrane order-sensitive fluorescent probe, di-4-ANEPPDHQ (Molecular Probes, Life Technologies). This probe displays a fluorescent spectral blue-shift from 620 nm when incorporated into lipid bilayers with a low-membrane lipid order (liquid disordered phase, L_d_) to 560 nm when inserted into lipid bilayers with high-membrane lipid order (liquid-ordered phase, L_o_). After acquisition using confocal fluorescence microscopy of both disordered and ordered-phase fluorescence images, a new image, indicative of membrane lipid order, was obtained by calculating the GP value—a ratiometric measurement of fluorescence intensities for each pixel which is associated to membrane lipid order [33,67]. Larva staining was realized as previously described [33]. After staining, they were mounted in 80% glycerol-PBS solution for the observation with confocal fluorescence microscope LEICA DMI 6000 CS (Leica Microsystems, Wetzlar, Germany). Under excitation at 488 nm with an argon ion laser, ordered membrane images were acquired with a PMT range of 500–580 nm, whereas for disordered membrane images the PMT range was 620–750 nm (magnification ×400). Using Fiji imaging processing software (ImageJ, [103]) and the macro published by Owen et al. [67], GP images were generated according to the following calculation: GP = (I500–580 − I620–750)/(I500–580 + I620–750). In order to avoid potential variation due to the different batches of larva used or to different staining, for each experiment—one batch of zebrafish larvae/one staining procedure—GP values were expressed as the difference between individual larva GP value and the mean of GP found in control larvae (ΔGP) within the same experiment.

Lipid-raft spatial distribution: lipid rafts are specialized membrane microdomains that can be defined by their high membrane order. Therefore, they were highlighted by selecting pixels with high GP values in comparison to the mean GP found in the control condition. Overall, the range of ΔGP values for lipid rafts was 0.142 to 0.381 in comparison to the mean value found for all membranes in the livers of the control larvae in the experiment (ΔGP = 0). Visualization of the membrane area with high GP value reflects the membrane regions with a local high lipid-raft distribution suggesting a higher level of clustering. Using Fiji imaging processing software (ImageJ, [103]), pixels with a high density were selected in GP images, which highlighted high-ordered membrane domains in yellow through the membrane area of the liver cells. Images presented are pseudo-colored GP images in which ΔGP values are indicated on a colour scale [67].

### 4.6. Statistical Analysis

All values were presented as mean ± SEM (standard error of the mean) from at least three independent experiments. Multiple comparisons among groups were performed using one-way analysis of variance (ANOVA) followed by a Newman–Keuls post-test. To evaluate the effect of the HFD diet, a one-tailed Student’s *t*-test was performed. All statistical analyses were performed using GraphPad Prism5 software (GraphPad Software, San Diego, CA, USA). Differences were considered significant when *p* < 0.05.

## Figures and Tables

**Table 1:** List of primers used for RT-qPCR experiments.

Gene	Official Full Name	Accession Number	Forward Primer	Reverse Primer
*actb2*	Actin, beta 2	NM_181601.4	5′-TTCTCTTAAGTCGACAACCCCC-3′	5′-TACCAACCATGACACCCTGAT-3′
*18s*	-	NR_145818.1	5′-TTACCCCAGGCTCGGAAAAC-3′	5′-CGGGAAGGTCTTTGAACCCA-3′
*gapdh*	Glyceraldehyde-3-phosphate dehydrogenase	NM_001115114.1	5′-GAGGCTTCTCACAAACGAGGA-3′	5′-TGGCCACGATCTCCACTTTC-3′
*crp*	C-reactive protein	NM_001045860.1	5′-CATTAGAGGCTACCGAAGGTTT-3′	5’-GACTCAGGGGTTTTTCAGGATA-3′
*nfkb3 (rela)*	Nuclear factor kappa B	NM_001001839.2	5′-CAACGACACCACGAAAACG-3′	5′-CGTCAGGAATCTTGAATGGGT-3′
*il1b*	Interleukin 1β	NM_212844.2	5′-GAACAGAATGAAGCACATCAAACC-3′	5′-ACGGCACTGAATCCACCAC-3′
*il6*	Interleukun6	NM_001261449.1	5’-TCAACTTCTCCAGCGTGATG-3′	5’-TCTTTCCCTCTTTTCCTCCTG-3′
*casp3a*	Caspase 3a	NM_131877.3	5’-TCGGTTCTCGCTGTTGAAGG-3′	5′-GTCTCCGTATCCGCATGTCC-3′
*tgfb1a*	Transforming growth factor β 1a	NM_182873.1	5′-GGAAGGCAACACAAGGTGGA-3′	5′-GGCTTACTTATCAATCCCGACT-3′
*tfa*	Transferrin a	NM_001291499.1	5’-GAAAATCCCAGAGTCAGCCA-3’	5′-TTCATCTCCAACAGCCTTCC-3′
*zgc163022*	Ferric chelate reductase 1	NM_001089557.2	5’-CCCAGAGGCTGCTGTTTATT-3’	5′-GCCGTGATTAGGCATCATAGAG-3′
*nrf2a*	Nuclear factor (eruthroid-derived 2)-like2	NM_182889.1	5′-TCGGGTTTGTCCCTAGATG-3′	5′-AGGTTTGGAGTGTCCGCTA-3′
*gstp1*	Glutathione S-transferase pi	NM_131734.3	5′-ACACACTCACATACTTCGCA-3′	5′-GTCGCCCTTCATCCACTCTT-3′
*nqo1*	NADPH dehydrogenase, quinone 1	NM_001204272.1	5′-TCTGACAAAGAAAGGCTACAAAGTC-3′	5′-ATACACAAAGTGCTCGGGATT-3′

## Supplementary Materials

The following are available online at http://www.mdpi.com/2218-273X/8/2/26/s1, Figure S1: Protective effect of pravastatin against the toxicity induced by B[a]P/ethanol co-exposure in steatotic WIF-B9 cell line; Figure S2: mRNA expression of *cyp1a* after exposing HFD zebrafish larvae to B[a]P and ethanol with or without pravastatin; Supplementary Methodology: WIF-B9 cell culture and treatment and Toxicity evaluation.
