# Supplementary material for: Membrane Remodeling as a Key Player of the Hepatotoxicity Induced by Co-Exposure to Benzo[a]pyrene and Ethanol of Obese Zebrafish Larvae
Source: Biomolecules. 2018 May 14;8(2):26. doi: 10.3390/biom8020026 (PMC6023014; doi:10.3390/biom8020026)
Supplement: Supplementary file 1 [file biomolecules-08-00026-s001.pdf]

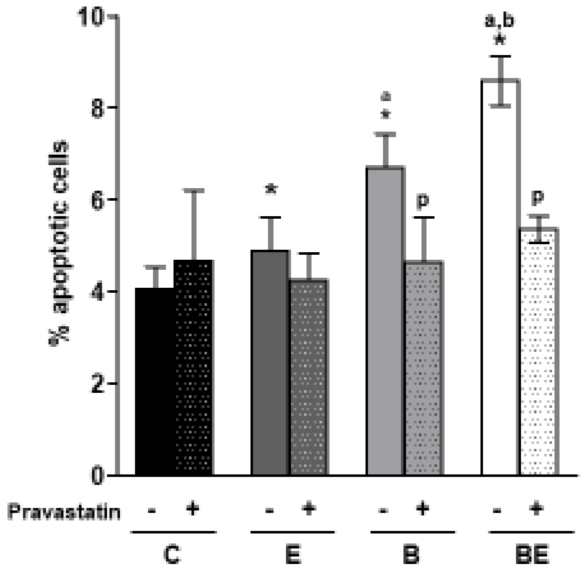

Supplementary Figure 1. Protective effect of pravastatin against the toxicity induced by B[a]P/ethanol co-exposure in steatotic WIF-B9 cell line. Steatotic WIF-B9 cells were untreated (C) or treated for an overall 5 days period to 5 mM ethanol (E), 10 nM B[a]P (B) or combination of both toxicants (BE). Apoptotic cell death with 10 μM pravastatin was determined by microscopic counting after hepatocyte staining with Hoechst 33242. Values are the mean ± SEM of three independent experiments. [*]Significantly different from steatosis control condition; [a]Significantly different from condition treated by ethanol only; [b]Significantly different from condition treated by B[a]P only; [p]Significantly different from cells treated by pravastatin.

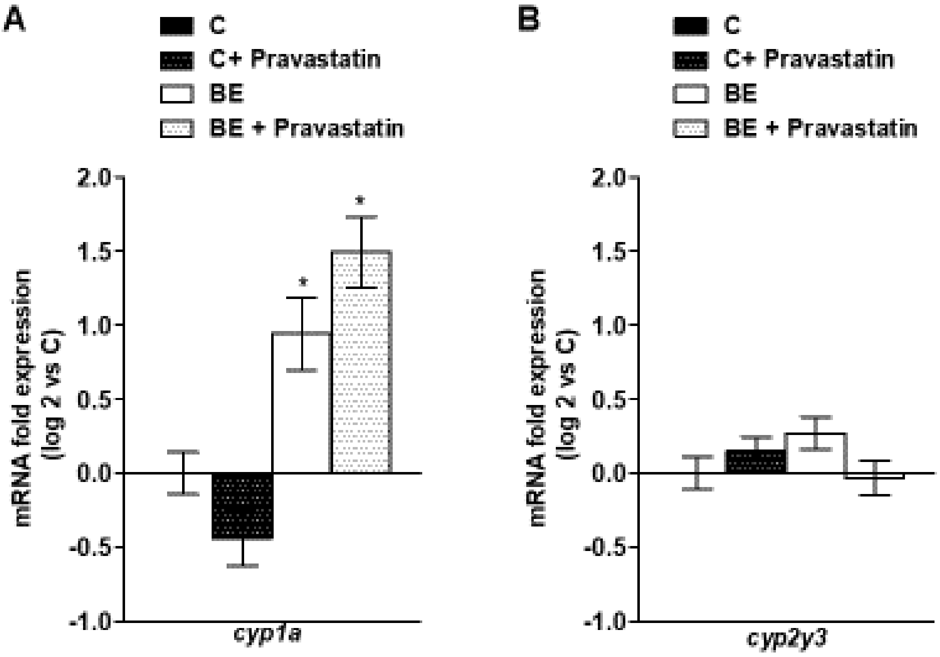

Supplementary Figure 2. mRNA expression of *cyp1a* and *cyp2y3* after exposing HFD zebrafish larvae to B[a]P and ethanol with or without pravastatin. mRNA expression of *cyp1a* ***(A)*** and *cyp2y3* ***(B)*** was evaluated by rt-qPCR . Zebrafish larvae were started to be fed with high-fat diet (HFD) from 4 dpf and from 5 dpf, they were either left untreated (C) or treated with co-exposure of 43 mM ethanol and 25 nM B[a]P (BE) until 12 dpf. Both conditions were also treated with 0.5µM pravastatin as quoted as (C±pravastatin) and (BE±pravastatin), respectively. Data are expressed relative to mRNA level found in HFD control larvae, set at 0 (log 2 change).Values are the mean ± SEM. [*]Significantly different from HFD control larvae. [P]Significant difference between larvae treated by pravastatin compared to untreated counterparts.

Methodology:

WIF-B9 cell culture and treatment: WIF-B9 is a hybrid cell line obtained by fusion of Fao rat hepatoma cells and WI-38 human fibroblasts [33,79–81]. The WIF-B9 cells were a generous gift from Dr Doris Cassio (UMR Inserm S757, Université Paris-Sud, Orsay, France). Cells were cultured in F-12 Ham medium with Coon's modification containing 5% FCS, 0.22 g/L sodium bicarbonate, 100 U/mL penicillin, 0.1 mg/mL streptomycin, 0.25 µg/mL amphotericin B, 2 mM glutamine, and supplemented with HAT (10 µM hypoxanthine, 40 nM aminopterin, 1.6 µM thymidine). WIF-B9 cells were seeded at 12.5 × 10^3 cells/cm^2; cells were cultured for 7 days until obtaining ~ 80% of confluence, before treatment.

The FA-albumin complex containing medium was prepared by FA saponification with a NaOH/ethanol solution at 70°C for 30 min. After ethanol evaporation under nitrogen, FA salts were solubilized in culture medium supplemented with 90 µM FA-free bovine serum albumin. Steatosis was induced by a two days treatment with a medium containing the FA/albumin complex composed of 450 µM oleic acid and 100 µM palmitic acid. Steatotic cells were then exposed or not for an overall

5 days period to the toxicants (10 nM B[a]P with or without 5 mM ethanol). Media and treatments with toxicants were renewed on day 3. For experiments with raft disrupting agent, cells were co-exposed with 10 μM pravastatin (Sigma-Aldrich) and toxicants—B[a]P and ethanol. Pravastatin was added 1 hour before the addition of toxicants.

Toxicity evaluation: WIF-B9 cells were tested for apoptotic cell death by fluorescence microscopic observation of cells stained with Hoechst 33342 (Life Technologies) and propidium iodide (Sigma-Aldrich). After each treatment, cells were stained with 10 μg ml^{-1} Hoechst 33342 and 10 μg ml^{-1} propidium iodide in the dark for 15 min at 37 °C. Cells were then examined by fluorescence microscopy (Olympus BX60; Olympus, Rungis, France). The total population was always more than 400 cells. Cells with condensed and/or fragmented chromatin were counted as apoptotic cells.

References

33. Podechard, N. et al. Zebrafish larva as a reliable model for *in vivo* assessment of membrane remodeling involvement in the hepatotoxicity of chemical agents: Zebrafish larva for assessing membrane remodeling by hepatotoxicants. *Journal of Applied Toxicology*, **2017**, *37*, 732–746.

79. Decaens, C.; Rodriguez, P.; Bouchaud, C.; Cassio, D. Establishment of hepatic cell polarity in the rat hepatoma-human fibroblast hybrid WIF-B9. A biphasic phenomenon going from a simple epithelial polarized phenotype to a hepatic polarized one. *J. Cell. Sci.* **1996**, *109*, 1623–1635.

80. Biagini, C. et al. Cytochrome P450 expression-induction profile and chemically mediated alterations of the WIF-B9 cell line. *Biol. Cell* **2006**, *98*, 23–32.

81. McVicker, B. L., Tuma, D. J., Kubik, J. L., Tuma, P. L. & Casey, C. A. Ethanol-induced apoptosis in polarized hepatic cells possibly through regulation of the Fas pathway. *Alcohol. Clin. Exp. Res.* **2006**, *30*, 1906–1915.
